# Women

**Published:** 1893-12-23

**Authors:** 


### WOMEN.

**Chelsea Hospital for Women,** Fulham Road, S.W.—This hospital has 60 beds, and treats respectable poor women and ladies in reduced circumstances who are victims of ills peculiar to their sex. Contributing in-patients occupy separate wards, paying 10s. 6d. to 42s. a-week, according to their means. The hospital is entirely without endowment or reserve funds, and earnestly desires more annual subscriptions from women who have pity on their sex. Secretary, Mr. A. C. Davis; Matron, Miss Ada Plumb.

**Grosvenor Hospital for Women and Children,** Vincent Square, Westminster, S.W.—The Grosvenor Hospital is situated in a densely-populated district of Westminster, and the bulk of the out-patients is drawn from the poorer classes living in the neighbourhood. In-patients, however, are received from all parts of the country, and both residents and non-residents of London may well give the institution a generous support. The hospital has been lately enlarged to meet the increased demands for its benefits, and the current annual expenditure is consequently increased. Secretary, A. S. Harvey; Matron, Miss K. Hughes.

**New Hospital for Women,** 144, Euston Road, N.W. —The principal feature of the hospital is that it enables poor women to consult qualified physicians of their own sex; and this boon has been so highly appreciated that the work begun in 1872, with ten beds, has increased to such an extent that a new hospital, with 42 beds, was opened in 1891 for the reception of in-patients. The beds have been continuously occupied ever since, and numbers are waiting for admission. Secretary, Miss M. M. Bagster.

**Samaritan Free Hospital for Women and Children,** Marylebone Road, N.W.—Some £7,000 per annum is required to maintain this hospital, of which only £1,750 can be relied upon in annual subscriptions; for the rest it has to depend entirely on donations—a most uncertain source of income—bequests, and awards from the Hospital Sunday Fund and Hospital Saturday Fund, &c. The committee would therefore like to see a great addition to the list of annual and life subscriptions, and earnestly appeal for Christmas and New Year's gifts. Bankers, Scott and Co., 1, Cavendish Square, W. Secretary, Mr. G. Scudamore; Matron, Miss Butler.

**The Hospital for Women,** Soho Square, W.—This institution claims the distinction of being the first established of its speciality, having been founded in the year 1842 exclusively for the treatment of diseases peculiar to women. There are 64 beds in constant use, and funds for their maintenance are much needed. During the past three years a large proportion of the income of the hospital has been derived from legacies; but as this source is always a very precarious one, the committee very earnestly appeal for additional annual subscriptions in order that the reliable income of the charity may be raised to an amount more commensurate with its requirements. Secretary, Mr. David Cannon; Matron, Miss Squier.

**The Royal Hospital for Children and Women,** Waterloo Bridge Road, S.E.—This hospital stands in the midst of the densest poverty—indeed, with the single exception of St. George's-in-the-East, the population is more crowded than that of any other portion of London, being 216 persons to the acre. This year the demand on the beds has been exceptionally high; the income of the year is insufficient to enable the hospital to continue to keep open all the wards unless further help is obtained. A sum of £1,000 is wanted, and that immediately, if this valuable institution is adequately to perform its good work. Secretary, Mr. R. G. Kestin; Matron, Miss Mary Mocatta.

